# HPV16 E6 and E7 Oncoproteins Stimulate the Glutamine Pathway Maintaining Cell Proliferation in a SNAT1-Dependent Fashion

**DOI:** 10.3390/v15020324

**Published:** 2023-01-24

**Authors:** Yunuen Ortiz-Pedraza, J. Omar Muñoz-Bello, Lucio Antonio Ramos-Chávez, Imelda Martínez-Ramírez, Leslie Olmedo-Nieva, Joaquín Manzo-Merino, Alejandro López-Saavedra, Verónica Pérez-de la Cruz, Marcela Lizano

**Affiliations:** 1 Unidad de Investigación Biomédica en Cáncer, Instituto Nacional de Cancerología, Mexico City 14080, Mexico; 2 Posgrado en Biología Experimental, DCBS, Universidad Autónoma Metropolitana-Iztapalapa, Mexico City 09340, Mexico; 3 Departamento de Neuromorfología Funcional, Dirección de Investigaciones en Neurociencias, Instituto Nacional de Psiquiatría Ramón de la Fuente Muñiz, Mexico City 14370, Mexico; 4 Cátedras CONACyT- Instituto Nacional de Cancerología, Mexico City 14080, Mexico; 5 Laboratorio de Neurobioquímica y Conducta, Departamento de Neuroquímica, Instituto Nacional de Neurología y Neurocirugía Manuel Velasco Suárez, Mexico City 14269, Mexico; 6 Departamento de Medicina Genómica y Toxicologóa Ambiental, Instituto de Investigaciones Biomédicas, Universidad Nacional Autónoma de Meóxico, Ciudad Universitaria, Mexico City 04510, Mexico

**Keywords:** HPV16 E6 and E7 oncoproteins, SNAT1 transporter, glutaminolysis

## Abstract

Persistent high-risk human papillomavirus infection is the main risk factor for cervical cancer establishment, where the viral oncogenes E6 and E7 promote a cancerous phenotype. Metabolic reprogramming in cancer involves alterations in glutamine metabolism, also named glutaminolysis, to provide energy for supporting cancer processes including migration, proliferation, and production of reactive oxygen species, among others. The aim of this work was to analyze the effect of HPV16 E6 and E7 oncoproteins on the regulation of glutaminolysis and its contribution to cell proliferation. We found that the E6 and E7 oncoproteins exacerbate cell proliferation in a glutamine-dependent manner. Both oncoproteins increased the levels of transporter SNAT1, as well as GLS2 and GS enzymes; E6 also increased LAT1 transporter protein levels, while E7 increased ASCT2 and xCT. Some of these alterations are also regulated at a transcriptional level. Consistently, the amount of SNAT1 protein decreased in Ca Ski cells when E6 and E7 expression was knocked down. In addition, we demonstrated that cell proliferation was partially dependent on SNAT1 in the presence of glutamine. Interestingly, SNAT1 expression was higher in cervical cancer compared with normal cervical cells. The high expression of SNAT1 was associated with poor overall survival of cervical cancer patients. Our results indicate that HPV oncoproteins exacerbate glutaminolysis supporting the malignant phenotype.

## 1. Introduction

Persistent high-risk *human papillomavirus* (HR-HPV) infection is the main risk factor for the development of different types of anogenital as well as head and neck cancers of which cervical cancer (CC) has the largest fraction attributable to HPV [1,2]. CC ranks fourth in mortality and incidence of neoplasms in women worldwide, particularly in developing countries [3,4]. 

The oncogenic potential of HPV is mainly attributed to the sustained expression of E6 and E7 oncoproteins, which affect a plethora of proteins involved in the acquisition of transforming characteristics that impact cell differentiation, inhibition of apoptosis, immortalization, metabolic reprogramming, and evasion of the immune response [5,6]. The most studied functions of the E6 and E7 oncoproteins lie in their ability to bind to and promote the degradation of p53 and pRb tumor suppressor proteins, respectively [7,8,9], leading to cell cycle dysregulation and resistance to apoptosis, which are key processes in carcinogenesis. 

Cancer cells reprogram their metabolic pathways to support the increased energy demand necessary for cell proliferation, which in CC is achieved by the actions of both E6 and E7 oncoproteins [10]. In cancer cells, glycolysis is the most widely used pathway to produce energy and drive different metabolic activities [11]. Other metabolic pathways are also altered during carcinogenesis or cancer maintenance, such as the glutamine pathway or glutaminolysis [12,13]. Glutamine is a carbon and nitrogen source with an essential role in cancer cell growth [14,15]. Glutamine participates in the synthesis of purines and pyrimidines and other amino acids, and it is a precursor for the synthesis of reduced glutathione (GSH) in the regulation of oxidative stress [16,17,18,19]. Therefore, glutamine is an important player in cancer metabolism. Under normal conditions, glutamine enters the cell via members of various families of transporters such as SLC38A, SLC7A, and SLC1A; the most characterized being SNAT1, ASCT2, LAT1, and xCT. Accumulated evidence reveals that glutamine transporters are dysregulated in various types of cancer [20,21,22,23,24]. Once in the cell, glutamine is deaminated in the mitochondria by glutaminase 1 and 2 (GLS and GLS2) enzymes, generating glutamate and ammonia [25]. Afterwards, glutamate is converted to α-ketoglutarate and ammonia by glutamate dehydrogenase (GLUD) [26]. Furthermore, glutamic-oxaloacetic transaminase 1 (GOT1) catalyzes the formation of glutamate from α-ketoglutarate in the cytosol, which is the reverse activity of aspartate aminotransferase (AST or GOT2) within the mitochondria [25]. In addition, glutamine can also be obtained from glutamate through glutamine synthetase (GS) activity [26].

Little information is available on the involvement of HPV16 in glutamine metabolism. Previous reports show that the HPV16 E7 oncoprotein increased glutamine and pyruvate uptake in NIH 3T3 cells with high glycolytic index, compared with those cells with low glycolytic index [27,28]. In A2780 ovarian cancer cells, ectopically expressed E6 increased glucose and glutamine uptake, which also promoted lactate and alanine secretion [29]. Moreover, an RNAseq analysis revealed low expression of the xCT transporter in HPV-positive head and neck squamous cell carcinomas (HNSCC) compared with HPV-negative HNSCC [30]. However, the regulation of elements involved in glutaminolysis by HPV oncoproteins remains elusive. Therefore, the aim of this study was to evaluate the role of the HPV16 E6 and E7 oncoproteins in the glutamine pathway and their contribution in supporting cervical cancer cell proliferation.

We found that proliferation is preferentially dependent on glutamine in cells expressing E6 and E7. Interestingly, E6 and E7 increase the levels of the SNAT1 transporter, as well as the GLS2 and GS enzymes. Furthermore, E6 also increases LAT1 transporter levels, while E7 increases ASCT2 and xCT. In some cases, the modulation of these proteins was also associated with increased gene expression. Consistently, the SNAT1 transporter protein was decreased in Ca Ski cells when E6 and E7 expression was abrogated. Finally, SNAT1 expression was found to be significantly higher in cervical cancer samples compared with normal cervical cells and was also associated with poorer overall survival in cervical cancer patients. 

## 2. Materials and Methods

### 2.1. Cell Culture 

C-33 A stably transfected cells harboring HPV16 E6 and E7 coding sequences cloned into the pXFLAG-CMV™-10 expression vector (Sigma Aldrich, Tokyo, Japan) were used. For protein identification, E6 and E7 genes were fused to Flag and HA-tag sequences, as previously described [31]. Stably transfected cells were grown in Eagle’s Minimum Essential Medium (EMEM) (ATCC) supplemented with 10% fetal bovine serum (FBS) and 2 g/L of Geneticin 418 (ChemCruz, Santa Cruz, CA, USA). Experimental groups of stably transfected cells were defined as EV (Empty Vector), E6 (HPV16 E6), or E7 (HPV16 E7). HPV16-positive Ca Ski cells were maintained in Roswell Park Memorial Institute (RPMI) 1640 Medium (Gibco, Thermo Fisher, Waltham, MA, USA) supplemented with 10% FBS. Cell cultures were maintained in a humified incubator at 37 °C in a 5% CO_2_ atmosphere. 

### 2.2. Proliferation Assays 

Stably transfected C-33 A cells were seeded in 60 mm dishes. After 24 h, cells were harvested and re-seeded in a 96-well plate and incubated with different treatments for 24 h, 48 h, and 72 h. Cells were grown with Dulbecco’s Modified Eagle’s Medium (DMEM) (Gibco, Thermo Fisher, Waltham, MA, USA) lacking D-glucose, L-glutamine, phenol red, and sodium pyruvate. Where indicated, the medium was supplemented with 4.5 g/L of glucose solution (Gibco, Thermo Fisher, Waltham, MA, USA) and/or 2 mM of L-glutamine solution (Gibco, Thermo Fisher, Waltham, MA, USA). Then, MTS (3-(4,5-dimethylthiazol-2-yl)-5-(3-carboxymethoxyphenyl)-2-(4-sulfophenyl)-2H-tetrazolium, inner salt) assay was performed using CellTiter 96 AQueous One Solution Cell Proliferation Assay Kit (Promega, Madison, WI, USA) according to the instructions indicated by the manufacturer.

For cell density evaluation, cells were fixed with PBS/formalin (10%) for 30 min at room temperature while shaking. Cells were then stained with crystal violet solution for 20 min. After several washes, the dye was eluted using PBS/acetic acid (10%) and absorbance was measured at 490 nm. Data were graphed to determine the percentage of cell proliferation for each tested condition.

### 2.3. Reverse Transcription Quantitative Polymerase Chain Reaction (RT-qPCR) 

Cells were seeded in 60 mm culture dishes and total RNA extraction was performed using the RNeasy Mini kit (Qiagen, Hilden, Germany). Isolated RNA was treated with DNAse Free DNA removal kit (Thermo Fisher Scientific, Waltham, MA, USA) and then 1 µg of RNA was reverse transcribed with random hexamers utilizing the High-Capacity cDNA Reverse Transcription Kit (Applied Biosystems, Foster City, CA, USA). Primers used for the amplification of each transcript are shown in the Supplementary Materials, Table S1. SYBR select Master Mix (Applied Biosystems, Foster City, CA, USA) was utilized for qPCR reactions. The results are presented as relative quantification using the ^∆∆^Ct method.

### 2.4. Western Blotting

Cells were cultured in 60 mm dishes. After 24 h, cells were lysed using 100 µL of RIPA lysis buffer (50 mM Tris-HCl, pH 7.4, 150 mM NaCl, 1% NP-40, 0.25% Sodium Deoxycholate, 1% SDS and protease inhibitor cocktail (Roche, Basel, Switzerland)). Total cell protein extracts (20 µg) were analyzed by 10% and 12% SDS-PAGE gels and transferred onto a 0.22 µm nitrocellulose membrane (Bio-Rad, Hercules, CA, USA). A solution of 10% skimmed milk in TBS-0.1% Tween 20 was used to block membranes during 1 h at room temperature. Further, membranes were incubated overnight with the appropriate primary antibody diluted in 2.5% milk/0.1% Tween 20/PBS. 

Primary antibodies included anti-GLS2 (Invitrogen, Waltham, MA, USA); anti-SLC7A5 (Invitrogen, Waltham, MA, USA); anti-xCT (Cell signaling); anti-ASCT2 (Cell signaling, Danvers, MA, USA); anti-GLS1 (Cell signaling); anti-SNAT1 (Cell signaling); anti-GS (Abcam, Cambridge, UK); anti-p53 (Santa Cruz Biotechnology, Santa Cruz, CA, USA); anti-Myc (Abcam), anti-HA-tag (Cell signaling); and anti-α-Tubulin (Cell signaling). Membranes were washed thrice with TBS-0.1% Tween 20 and incubated with HRP-conjugated secondary anti-mouse or anti-rabbit antibody in a dilution 1:10000 dilution (Santa Cruz Biotechnology). Proteins were visualized through film-based imaging utilizing the Clarity Western ECL (Bio-Rad). Western blots were performed at least thrice to ensure result reproducibility.

### 2.5. Metabolic Measurements

Stably transfected C-33 cells were seeded for 24 h in DMEM medium with 10% FBS. Later, cells were thoroughly washed with PBS to ensure removal of glutamine or other metabolites, excluding possible cross contaminations. Cells were then starved for two hours in DMEM medium without glucose or glutamine. Afterward, 2 mM of glutamine was added to cell cultures for subsequent measurements by HPLC at different times (5 min, 15 min, and 30 min). The group of cells that were not treated with glutamine was considered as time 0. Glutamine and glutamate levels were determined by HPLC (high-performance liquid chromatography) using a fluorescence detector model S200 (PerkinElmer, Waltham, MA, USA) at excitation wavelength 232 nm and emission wavelength 455 nm. For such purposes, samples were previously lysed with 85% methanol, centrifuged at 12500 rpm for 15 min at 4 °C, and supernatant was obtained. Afterward, 10 µL of supernatant was treated with OPA reagent (containing 25 mg OPA + 625 µL methanol + 5.6 mL of 0.3 M borate buffer (pH 9.5) + 25 µL 2-mercaptoethanol) and eluted through a methanol gradient at a flow rate of 1.5 mL/min. The mobile phase containing 0.29% acetic acid (JT Baker, Gallade Chemical, Santa Ana, CA, USA) and 1.5% tetrahydrofuran (Mallinckrodt, Blanchardstown, Dublin, Ireland), pH 5.9, was added. The derivatives were separated through a PROTECOL C18G125 column of 5 µm particle size, 150 mm × 4.6 mm (Alltech, Nicholasville, KY, USA). The retention time for glutamate was ~3.6 min and for glutamine ~6.4 min. The results are presented as nmoles of glutamine/mg of protein and nmoles of glutamate/mg of protein.

### 2.6. HPV16 E6/E7 and SNAT1 siRNA Knock Down 

Ca Ski cells were seeded in 60 mm dishes and transfected with small interfering RNAs targeting siE6/E7 (Dharmacon, Lafayette, CO, USA) or siSNAT1 (Santa Cruz, Bio, Santa Cruz, CA, USA). As the control, siLuc (Dharmacon, Lafayette, CO, USA) was used. Transfections were performed with 9 μL of Lipofectamine™ RNAiMAX (Invitrogen, Waltham, MA, USA) according to the manufacturer’s instructions. After 72 h, cells were either fixed for fluorescence analyses or harvested to obtain protein extracts using RIPA buffer. Protein levels were evaluated by Western blot, the location of each protein was analyzed by immunofluorescence, and gene expression was evaluated by qRT-PCR, as indicated.

### 2.7. Immunofluorescence and Cell Imaging 

Ca Ski cells were seeded on slides in 60 mm dishes and transfected with the indicated siRNA. After 72 h, cells were fixed with 3.7% paraformaldehyde in PBS for 15 min and permeabilized with 0.04% NP-40/PBS. Cells were incubated with 0.1M Glycine and 5% BSA solution for 1h at room temperature for blocking purposes. Then, cells were incubated overnight at 4 °C with anti-p53 (Santa Cruz Biotechnology) and anti-SNAT1 (Cell signaling) antibodies. After several washes with PBS 1×, cells were incubated with Alexa fluor 488 donkey anti-mouse (Invitrogen, Waltham, MA, USA) and Alexa fluor Rhodamine Red-X goat anti-rabbit (Invitrogen, Waltham, MA, USA), respectively, for 2 h at 4 °C. Slides were washed and mounted with Vectashield mounting medium containing DAPI (Vector Laboratories, Newark, CA, USA). Slides were visualized with a confocal microscope (Zeiss LSM 710 DUO, Oberkochen, Baden-Wurttemberg, Germany) with lasers giving excitation lines at 488 and 594 nm. Around twenty fields were observed for each treatment and representative images were acquired. Data of three independent experiments were collected with a 63X objective oil immersion lens. In addition, the same protocol was carried out to evaluate SNAT1 in C-33 A stably transfected cells. 

### 2.8. Cervical Samples

A total of 23 cervical cancer samples were obtained from the Tumor Biobank of the Instituto Nacional de Cancerología of Mexico City (INCan) and 18 normal HPV-negative cervical samples were kindly provided by the Instituto Nacional de Salud Pública (INSP). Samples were subjected to RNA extraction for qPCR analysis to determine SNAT1 expression. The protocol was approved by the Institutional Scientific and Ethical committees of INCan, ref. (017/007/IBI) (CEI/1144/17). All patients whose samples were utilized in this study agreed to participate and signed the informed consent form.

### 2.9. Statistical Analysis 

All experiments were performed three times and data were depicted as the mean ± standard error of the mean (SEM). To determine significant statistical changes in the experimental conditions compared with the control, we used a Student’s *t*-test and a *p* value of <0.05 was considered statistically significant.

## 3. Results

### 3.1. HPV16 E6 and E7 Oncoproteins Promote Glutamine-Dependent Proliferation

**Figure 1:**
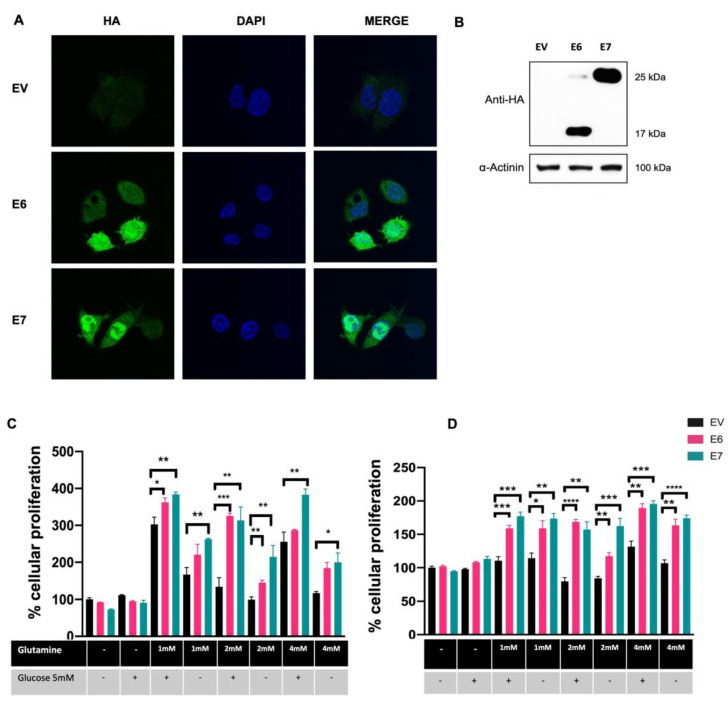
HPV16 E6 and E7 promote glutamine-dependent proliferation in C-33 A cells stably transfected with the HPV-16 E6 and E7 oncoproteins. (**A**) Detection of E6 and E7 HA-tagged oncoproteins in C-33 A cells (green) and DAPI-stained cell nuclei are shown in blue. (**B**) Immunodetection of E6 and E7 HA-tagged proteins in C-33 A cells. In E6-expressing C-33 A cells, the full length and the spliced E6* isoform are also detected. (**C**) MTS and (**D**) Crystal Violet assays were performed to assess glutamine-dependent cell proliferation. Cells were grown in media supplemented with different concentrations of glutamine (1 mM, 2 mM, and 4 mM) and glucose (5 mM) as indicated. Cells harboring E6 (pink bars), E7 (green bars), or the control EV (black bars) are depicted. Graphs show the data as the mean and ± SEM of three independent experiments. Statistical analysis was performed using Student’s *t*-test to analyze significant values when comparing E6 and E7 with EV within each group, as indicated. * *p* < 0.05, ** *p* < 0.01 and *** *p* < 0.001 and **** *p* < 0.0001.

C-33 A cells stably transfected with plasmids expressing HPV-16 E6 and E7 and Empty Vector (EV) were used as previously described [31]. As expected, detection of exogenous HA-tagged HPV16 E6 and E7 proteins was successfully achieved by immunofluorescence (Figure 1A) exhibiting mostly a nuclear localization; although, viral oncoproteins are also observed in the cytoplasm. Western blot assays (Figure 1B) show HA-tagged E6 and E7 proteins, where E6* is the most abundant isoform in E6-expressing cells. 

To demonstrate glutamine-dependent proliferation in cells expressing E6 and E7 oncoproteins, cells were supplemented with different concentrations of glutamine (0 mM, 1 mM, 2 mM, and 4 mM) in the presence or absence of 5 mM of glucose. Cell proliferation was assessed 72 h after glutamine exposure using MTS or Crystal Violet assays. As shown in Figure 1C, the MTS assay revealed no differences in cell proliferation in cells expressing E6 and E7 compared with those with EV, when glutamine and glucose were absent. Surprisingly, the same effect was observed even when the growth medium was supplemented with 5 mM glucose alone. After glutamine addition, increased proliferation was observed in all tested groups when evaluated in MTS assays, being significantly higher in cells expressing E6 and E7. Importantly, cells expressing E7 treated with 1 mM, 2 mM, or 4 mM glutamine alone had a significant increase in proliferation, relative to the control group with the same treatment (1.56-, 2.16-, 1.70-fold change, respectively). Similarly, E6-expressing cells treated with 1 mM, 2 mM, or 4mM glutamine alone presented an increase in proliferation, relative to the control group with the same treatment (1.31-, 1.46-, 1.57-fold change, respectively), however, only 2 mM glutamine treatment showed significance. Interestingly, when cells were incubated with 5 mM glucose plus glutamine, proliferation was increased when compared with cells without glutamine/glucose treatment or glucose alone. Particularly, in the group treated with 2 mM glutamine plus glucose, a significant difference was observed in cells expressing E6 and E7 compared with EV (2.42- and 2.33-fold change, respectively).

The crystal violet assay depicted in Figure 1D shows similar behavior to that obtained using the MTS assay, where cells with E6 and E7 increased proliferation in the presence of glutamine alone at 1 mM (1.39- and 1.52-fold change, respectively); 2 mM (1.39- and 1.92-fold change, respectively); and 4 mM (1.53- and 1.62-fold change, respectively). However, no additional advantage in this effect was identified by adding glucose. According to the results obtained with the MTS assays, no changes were observed in the absence of glutamine and glucose, nor in cells treated with glucose alone. These results strongly suggest that E6 and E7 oncoproteins promote the use of the glutamine pathway as the primary energy source to support cell proliferation. 

### 3.2. Intracellular Glutamine and Glutamate Increase in the Presence of HPV16 E6 Oncoprotein

To characterize the glutaminolysis profile of C-33A cells expressing EV, E6, or E7, intracellular glutamine and glutamate metabolites were measured through HPLC assay. Cells were fasted for two hours in medium deprived of glutamine and glucose. Subsequently, EV, E6, and E7 groups were exposed to 2 mM glutamine at different times (0 min, 5 min, 15 min, and 30 min) and HPLC assays were performed. The group of cells that were not treated with glutamine was considered as time 0.

**Figure 2:**
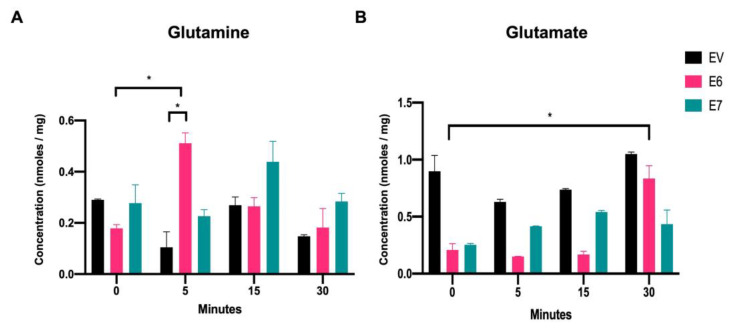
Intracellular glutamine and glutamate increase in the presence of the HPV16 E6 and E7 oncoproteins. HPLC analysis of (**A**) intracellular glutamine and (**B**) intracellular glutamate in C-33 A cells stably transfected with EV (black bars), E6 (pink bars), or E7 (green bars). The data were evaluated at 0 min, 5 min, 15 min, and 30 min after adding glutamine to the media. Statistical analysis using Student’s *t*-test was performed to obtain significant values when comparing cells harboring E6 and E7 with the EV group, as indicated. * *p* < 0.05 is considered to be significant.

Figure 2A shows that after 5 min of treating the cells with glutamine, a significant increase in intracellular glutamine concentrations was achieved in cells expressing the E6 oncoprotein, compared with with those harboring EV (4.91-fold increase), and an increase was also observed compared with E6 expressing cells at time 0 (2.85-fold change). At 15 min and 30 min, intracellular glutamine values in cells expressing E6 return to levels similar to those detected for EV in each group. These results suggest that the HPV16 E6 oncoprotein increases intracellular glutamine immediately after exposure to glutamine. Meanwhile, in cells with E7 expression, an increase in intracellular glutamine was observed after 15 min of glutamine treatment (1.63-fold increase), which was related to the EV condition and to cells expressing E7 at time 0 (1.58-fold); however, even when an increasing trend was observed, the results were not significant. 

When evaluating the intracellular glutamate concentrations upon exposure to glutamine (Figure 2B), it was observed that cells expressing E6 or E7 had lower glutamate levels than those with EV at time 0. This could be partly explained if cells with the viral oncoproteins have accelerated glutamate consumption which deserves further study. Upon addition of glutamine, E6-expressing cells showed a significant increase in the intracellular glutamate concentration after 30 min (4-fold change, relative to time 0). Furthermore, glutamate levels in EV cells remained similar in all tested conditions. Interestingly, cells expressing E7 showed an increase in intracellular glutamate levels after 5 min, 15 min, and 30 min (1.64-, 2.13-, and 1.71-fold change, respectively), compared with E7-expressing cells at time 0, although not significant. These results suggest that in E6- and E7-containing cells, glutamate is derived from the deamination of glutamine along glutaminolysis. 

### 3.3. SNAT1 Glutamine Transporter Expression and Protein Levels Increase in the Presence of HPV16 E6 and E7 Oncoproteins 

**Figure 3:**
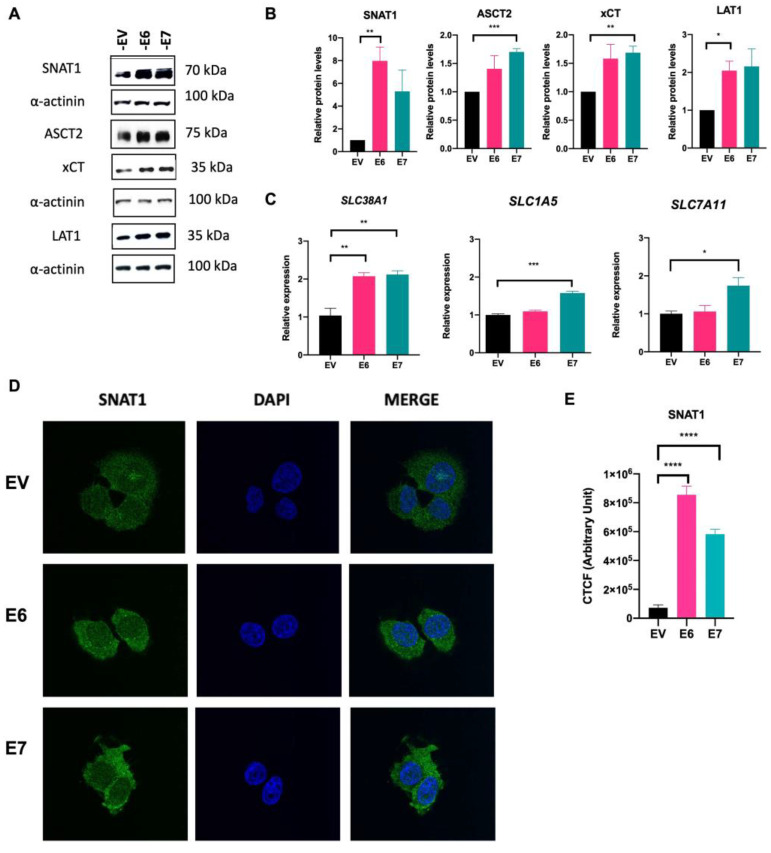
SNAT1 transporter is increased in the presence of HPV16 E6 and E7 oncoproteins. Protein levels and gene expression of the glutamine transporters were analyzed by Western blot and qPCR assays, respectively, in C-33 A cells harboring EV (black bars), E6 (pink bars), and E7 (green bars). (**A**) Representative immunoblots of SNAT1, ASCT2, xCT, and LAT1 are depicted. As loading control α-actinin was used. (**B**) Relative protein levels were obtained from densitometric analysis of immunoblots. (**C**) Relative expression analysis of SLC38A1, SLC1A5, and SLC7A11 genes was assessed by qPCR. (**D**) Stably transfected C-33 A cells were immunostained with anti-SNAT1 antibody (green) and DAPI for nuclear visualization (blue). (**E**) Corrected total cell fluorescence (CTCF) was obtained for SNAT1. Images were visualized with a 63x objective oil immersion lens. Data from three independent experiments were collected and plotted showing the mean and ±SEM. Student’s *t*-test was performed to obtain statistical differences between the E7 and E6 groups compared with empty vector values (EV). * *p* < 0.05, ** *p* < 0.01, *** *p* < 0.001, and **** *p* < 0.0001 values are represented as indicated.

To determine whether E6 and E7 oncoproteins affect the expression and protein content of glutamine-related transporters, Western blot, qPCR, and immunofluorescence assays were performed. As shown in Figure 3A and B, a remarkable increase in the protein levels of SNAT1 and LAT1 transporters was observed in the presence of E6, relative to the EV control group (7.97- and 2.04-fold change, respectively). Meanwhile, E7 significantly increased the levels of the ASCT2 and xCT transporter proteins (1.70- and 1.68-fold, respectively). 

Furthermore, qPCR assays (Figure 3C) revealed that E6 and E7 induced the expression of the SLC38A1 gene (SNAT1) (2.07- and 2.11-fold change, respectively). Moreover, E7 significantly upregulated gene expression of SLC1A5 (ASTC2) (1.57-fold change) and SLC7A11 (xCT) (1.74-fold change). Interestingly, immunofluorescence assays shown in Figure 3D and E confirm overexpression of SNAT1 in E6- and E7-expressing cells, compared with the EV control. Taken together, these results indicate that E6 and E7 oncoproteins preferentially regulate SNAT1 as a key glutamine transporter.

To support these results, we evaluated another stably transfected clone of C-33 A cells expressing E6 or E7 (E6-Clone 2 and E7-Clone 2) and found increased SNAT1 protein in relation to the control (EV-Clone 2) (1.72-, 2.31-fold change, respectively) (Supplementary Materials, Figure S1).

### 3.4. HPV16 E6 and E7 Modify Glutaminolysis-Related Components 

**Figure 4:**
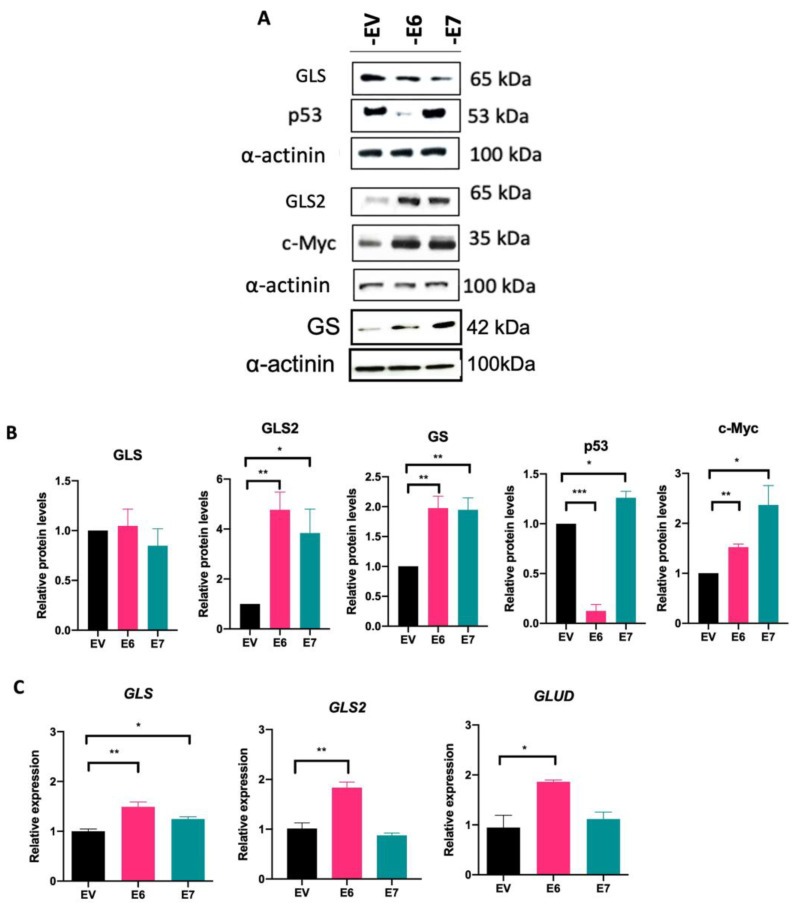
HPV16 E6 and E7 oncoproteins alter components of the glutamine pathway. (**A**) Immunodetection of GLS, GLS2, GS, p53, and c-Myc proteins in cells expressing E6 and E7 and (**B**) densitometric analysis. (**C**) Transcript levels of GLS, GLS2, and GLUD analyzed by qPCR. Data from three independent experiments were collected and plotted showing the mean and ±SEM. Statistical analysis was performed using Student’s *t*-test to decipher significant values * *p* < 0.05, ** *p* < 0.01, and *** *p* < 0.001 vs the values of the empty vector. As a loading control, α-actinin was used in the Western blot assay and 18S was used for normalization in the qPCR assays.

As we previously demonstrated that the HPV16 E6 and E7 oncoproteins promote upregulation of transporters that mediate glutamine uptake, we evaluated key components of the glutamine pathway. GLS, GLS2, and GS protein levels were assessed by Western blot. In Figure 4A,B, GLS protein levels do not change in cells expressing E6 and E7, compared with EV cells. Surprisingly, when evaluating GLS2 and GS, protein levels increased in the presence of E6 (4.76- and 1.97-fold change, respectively) and E7 (3.83- and 1.94-fold change, respectively). We also evaluated p53 and c-Myc proteins since they are known to be transcriptional regulators of glutaminolysis. As expected, p53 protein is virtually eliminated in the presence of E6, while an increase in this protein was observed in cells containing E7, which is consistent with previously reported data [32]. In addition, there is a significant increase in c-Myc protein levels in cells expressing E6 (1.52-fold change) or E7 (2.36-fold change). 

The transcript levels of GLS, GLS2, and GLUD were analyzed by qPCR (Figure 4C). The E6 and E7 oncoproteins promote a significant increase in GLS expression compared with EV (1.37- and 1.26-fold change, respectively), while GLS2 and GLUD expression only augmented in the presence of E6 (1.88- and 1.86-fold change, respectively). Taken together, our results demonstrate that both viral oncoproteins regulate the glutamine pathway by enhancing amino acid transporters and downstream components involved in this pathway. 

### 3.5. SNAT1 Transporter Expression and Protein Levels Are Reduced in Ca Ski E6/E7-Silenced Cells

**Figure 5:**
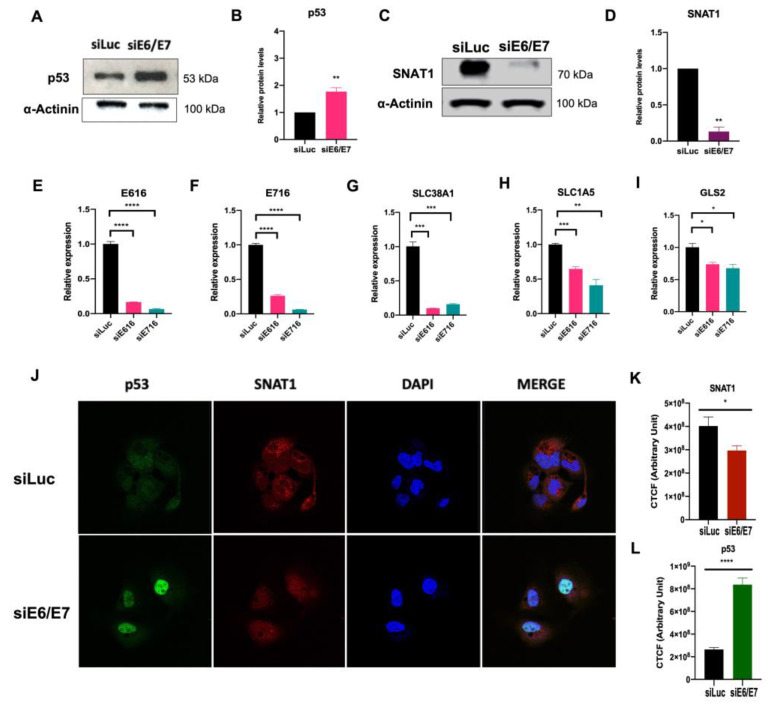
SNAT1 transporter is affected by the silencing of E6 and E7 expression in HPV-positive Ca Ski cells. Ca Ski cells were transfected with siLuc (control), siRNAE6/E7, siE6, or siE7. After 72 h of treatment, proteins were analyzed through Western blot. (**A**, **B**) Protein levels of p53 were restored in the absence of E6 and E7. (**C**, **D**) SNAT1 protein transporter levels decreased after ablation of E6/E7, as loading control α-actinin was used in Western blot. (**E**) Gene expression of HPV16 E6 and (**F**) E7, (**G**) SLC38A1 (SNAT1), (**H**) SLC1A5 (ASCT2), and (**I**) GLS2 decreased after ablation of E6 or E7. The 18S gene was used for normalization in qPCR assays. (**J**) SNAT1 and p53 levels were analyzed through immunofluorescence. Restoration of p53 (shown in green) and a decrease in SNAT1 protein levels (shown in red) were observed in E6/E7-silenced cells. Images were collected with a 63× objective oil immersion lens. (**K**) CTCF arbitrary fluorescence units were obtained for quantification of SNAT1 and (**L**) p53 protein levels. Data from three independent experiments were collected and plotted showing the mean and ±SEM. Statistical analysis was carried out using Student’s *t*-test to obtain significant values. * *p* < 0.05, ** *p* < 0.01, and **** *p* < 0.0001 vs. siLuc.

Since ectopically expressed E6 and E7 oncoproteins increased SNAT1, we were interested in evaluating the consequences of silencing E6 and E7 in Ca Ski cells, which endogenously contain HPV16 sequences and continuously express those oncoproteins. Ca Ski cells were transfected with small interfering RNAs (siRNA) targeting E6 mRNA (siE6), E7 mRNA (siE7), and E6/E7 mRNA bicistron (siE6/E7). An unspecific siRNA (siLuc) was used as a control. Figure 5E and F show that silencing of E6 and E7 expression was efficiently achieved. After 72 h, cell protein lysates were collected or fixed for immunodetection analysis by Western blot and immunofluorescence assays, respectively. As expected, when E6/E7 were knocked down in Ca Ski cells, a recovery in p53 protein levels was observed by Western blot (Figure 5A,B) and immunofluorescence (green) (Figure 5J,L). Interestingly, when evaluating the SNAT1 transporter by immunoblot as shown in Figure 5C,D and immunofluorescence (red) (Figure 5J,K ), SNAT1 levels were significantly decreased when E6/E7 expression was ablated. In addition, a significant decrease in the expression of SLC38A1 (SNAT1), SLC15A (ASCT2), and GLS2 was observed in cells where E6 or E7 expression was knocked down (Figure 5G–I, respectively). These data confirm that E6 and E7 alter elements of glutaminolysis, particularly the SNAT1 transporter, which may have an impact on the glutamine metabolism.

### 3.6. SNAT1 Transporter Partially Contributes to Ca Ski Cell Proliferation

**Figure 6:**
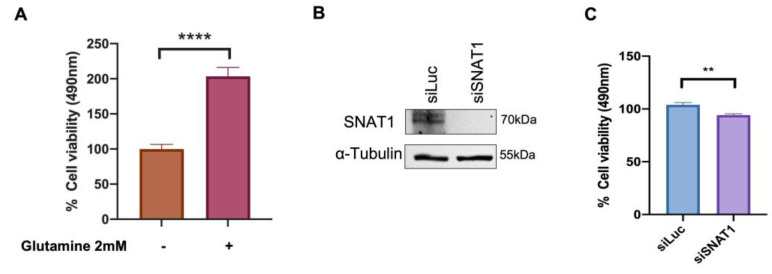
Ca Ski cell proliferation is increased in the presence of glutamine which is partially attributed to the SNAT1 transporter. (**A**) Ca Ski cells were grown in DMEM medium supplemented with 1 mM glucose and in the presence or absence of 2 mM glutamine as indicated. MTS proliferation assay was performed after 72 h. (**B**) SNAT1 silencing was evaluated through immunoblot in cells transfected with siLuc or siSNAT1; α-tubulin was used as loading control. (**C**) Proliferation was measured by MTS assay in Ca Ski cells doubly transfected with siSNAT1 and siLuc was used as the control. Graphs show data as the mean and ± SEM of three independent experiments. Statistical analysis was performed using Student’s *t*-test to analyze the significant values when comparing with the indicated groups, significance is represented as ** *p* < 0.01 and **** *p* < 0.0001.

To determine whether glutamine supports Ca Ski cell proliferation, MTS assays were performed in cells supplemented with low concentrations of glucose (1 mM) in the presence or absence of 2 mM glutamine, and assays were performed 72 h after treatments. As shown in Figure 6A, when Ca Ski cells were treated with low glucose plus 2 mM glutamine, a significant 2-fold increase in cell viability was observed compared with Ca Ski cells exposed to low glucose alone. It is worth mentioning that when Ca Ski cells are treated with 2 mM glutamine in the absence of glucose at 72 h, proliferation is also increased compared with the absence of glutamine (data not shown), evidencing the glutamine requirement for successful growth.

Furthermore, to elucidate the specific involvement of the SNAT1 transporter in Ca Ski cell proliferation, cells were transfected with siRNA targeting SNAT1 and siLuc (as control). As previously reported by Liu X. et al. 2020 [33], a double knockdown of SNAT1 was performed. Ca Ski cells were transfected again 72 h after the first transfection. Then, 24 h after the second transfection, Ca SKi cell proliferation was assessed using the MTS assay. SNAT1 protein levels were evaluated after 72 h of silencing and virtually no SNAT1 protein was detected (Figure 6B). A significant 8% reduction in proliferation was observed when SNAT1 was abolished, compared with the control (siLuc) (Figure 6C). These results support the idea that SNAT1 partially sustains cell proliferation of Ca Ski cells.

### 3.7. SNAT1 Expression Is Increased in Cervical Cancer Samples and Its High Expression Is Associated with a Poorer Prognosis in Cervical Cancer Patients

**Figure 7:**
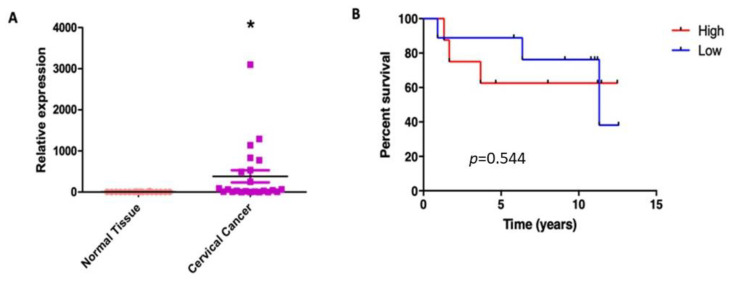
SNAT1 expression is increased in cervical cancer and its high expression is associated with worse prognosis. (**A**) SNAT1 expression was increased in cervical cancer samples (*n* = 23) compared with normal cervical samples (*n* = 18); * *p* < 0.05 represents significant differences in both groups. (**B**) Differences in the overall survival analysis according to the median expression of SNAT1, as high (*n* = 10) or low expression (*n* = 9) (*p* = 0.544). Low mRNA levels are represented by blue lines and high levels by red lines.

Our results suggest that HPV16 E6 and E7 induce the proliferation of cervical cancer cells partially dependent on the SNAT1 transporter. We wondered whether SNAT1 expression could be affected in cervical cancer and if it was also associated with patient clinical outcome. To assess differences in SNAT1 expression in cervical cancer samples compared with normal samples, RT-qPCR assays were performed. As shown in Figure 7A, SNAT1 expression was significantly increased in cervical cancer tissues compared with normal samples. Interestingly, as shown in Figure 7B, based on median expression, high SNAT1 expression in cervical cancer samples tends to be associated with poor overall survival (OS) although not significant (*p* = 0.544). These interesting results are consistent with those described in the Human Protein Atlas portal [34], where high SNAT1 expression exhibits low OS in patients with cervical cancer. However, a more robust study including a larger number of patient samples is needed to confirm such observations.

## 4. Discussion

An emerging feature of cancer cells is energetic metabolic reprogramming [35], which rewires metabolic pathways for different nutritional requirements to provide energy and building blocks to support several cellular processes associated with carcinogenesis, such as cell survival and exacerbated growth, among others [36,37,38]. 

Metabolic reprogramming of the glutamine pathway has been reported to promote pleiotropic functions in cells, including the synthesis of fatty acids, purines, and pyrimidines as well as redox homeostasis, which are also essential for the maintenance of cancer cell functions. Glutaminolysis has been found to be increased in different types of cancer including liver [39], glioma [40], lung [41], breast [42], and ovarian [43], among others. Therefore, the study of the elements involved in the glutamine pathway is of interest to understand the molecular mechanisms that underlie the cancerous process, in addition to exploring the potential use of these elements as therapeutic targets. However, little is known about the alterations that occur in glutamine metabolism in cervical cancer and the involvement of the HPV16 E6 and E7 oncoproteins in this process to maintain the malignant phenotype.

Previously, E6 and E7 oncoproteins were reported to induce metabolic reprogramming by affecting other metabolic pathways such as glycolysis, Krebs cycle, nucleotide synthesis, fatty acid synthesis, mitochondrial respiration, and autophagy [10,44]. In this study, we focused on elucidating the participation of HPV16 E6 and E7 oncoproteins in the regulation of the glutamine pathway in a cellular model of cervical cancer. We show that C-33 A cells, exogenously expressing HPV-E6 and -E7 oncoproteins, exhibit exacerbated proliferation in a glutamine-dependent manner and, surprisingly, no effect was observed when cells were exposed to glucose alone. These data consistently support the idea that HPV oncoproteins induce cell proliferation in a glutamine-dependent manner, being essential for maintaining oncogenic processes in HPV-related cancers. Similarly, Ca Ski cells, which contain HPV16 sequences, also showed increased proliferation in the presence of glutamine. It is worth mentioning that other amino acids, such as leucine and cysteine, enhance glutaminolysis [45,46]; therefore, their involvement in cell proliferation in HPV-related cancers cannot be ruled out and warrants further studies.

Comparably, it has been reported that in glutamine-deprived ovarian cancer cell lines (HEY, SKOV3, and IGROV-1) the addition of glutamine augments cell proliferation in a dose-dependent manner [43]. Moreover, it has been demonstrated that cancer aggressiveness correlates with glutamine-dependent proliferation. In breast cancer cell lines, it was observed that when cells were deprived of glutamine for 96 h, metastatic cells (MCF7 and MDA-MB231) substantially reduced their proliferation, while non-metastatic cells were not affected (MCF-10A and BT-20) [47].

Furthermore, by evaluating the participation of E6 and E7 in the glutamine/glutamate flux, we have shown that after exposing cells to 2 mM glutamine, E6 proteins increased intracellular glutamine concentrations after 5 min. Meanwhile, a trend towards increased intracellular glutamine in cells expressing E7 was observed at 15 min and 30 min. Likewise, E6 promotes accumulation of glutamate at 30 min, while an ascending behavior was detected in those cells with E7 expression at 5 to 30 min, although not significant. 

Consistent with our results, in a cell model of ovarian cancer, E6 expression increased glutamine consumption in A2780 cells [29]. In addition, HPV-16 E7 oncoprotein was reported to enhance glutamine uptake in two strains of NIH 3T3 cells with different metabolic characteristics, high- (hg-NIH) and low- (lg-NIH) glycolytic rate, where lg-NIH cells with E7 exhibited the highest consumption of glutamine and lower intracellular glutamate concentration [27]. It should be noted that these cells were treated with DMEM medium supplemented with 2 mM glutamine, which are similar conditions to those used in this work, where the culture medium lacked glucose. These results suggest that E6 and E7 oncoproteins can mediate glutamine/glutamate flux though alterations in glutamine transporters, facilitating intracellular glutamine access.

Several amino acid transporters that mediate glutamine uptake have been shown to be overexpressed in different types of cancer, including colon, breast, liver, lung, and osteosarcoma, among others [22,48,49,50,51,52]. This fact suggests that tumors strongly require glutamine intake to support cell survival and tumor growth. Since we found an increase in intracellular glutamine in cells expressing E6 and E7, we focused on evaluating the best-known glutamine transporters involved in glutamine uptake. Interestingly, we found that both E6 and E7 increased SNAT1 protein and transcript levels compared with EV cells (Figure 3). 

Furthermore, effects individually exerted by each oncoprotein were observed, where E6 increased LAT1 protein levels and E7 increased ASCT2 and xCT proteins and transcripts. To demonstrate the specific involvement of E6 and E7 oncoproteins in glutamine regulation in an HPV-positive cancer cell line, we knocked down the expression of E6 and E7 in Ca Ski cells. As expected, recovery of p53 in the nucleus was observed after E6/E7 ablation, compared with control cells. Interestingly, in Western blot and immunofluorescence assays (Figure 5), SNAT1 was significantly decreased when E6/E7 was knocked down. Furthermore, the expression of SNAT1, ASCT2, and GLS2 genes was also reduced. Taken together, these results suggest that E6 and E7 oncoproteins converge in modulating SNAT1, which may play a key role in glutamine uptake in cervical cancer, although other transporters, including ASCT2 and xCT, are also involved. 

Additionally, by evaluating downstream components of the glutamine pathway, E7 and E6 were found to increase GLS2 and GS protein content, demonstrating that there is a balance in glutamine and glutamate metabolic flux. It should be noted that both oncoproteins may favor the use of the GLS2 enzyme over GLS. Previous studies indicate that both isoforms may play oncogenic and anti-oncogenic roles depending on the type of cancer [53]. For example, in breast cancer, GLS2 amplification or overexpression is associated with acquisition of a malignant phenotype and poor prognosis [54,55]. Moreover, GLS2 is known to be transcriptionally regulated by p53 [56,57], promoting glutamate synthesis and glutathione homeostasis [58]. As expected, in C-33 A cells expressing E6, p53 levels are overwhelmingly decreased, and despite this, GLS2 levels are increased. This could be explained if GLS2 expression could also take place in a p53-independent context, for example, being regulated by p63 [59]. Moreover, c-Myc has been shown to promote GLS expression in different cancer cell lines [60]. We demonstrated a significant increase in c-Myc protein in E6 and E7 expressing cells, which correlates with increased GLS expression, suggesting that viral oncoproteins upregulate c-Myc, affecting GLS transcription. However, no differences in GLS protein content were observed, possibly due to other GLS regulation mechanisms not explored in this work.

As we demonstrated that HPV-16 E6 and E7 oncoproteins consistently increase SNAT1 protein levels, our research focused on elucidating the specific involvement of SNAT1 in glutamine-dependent proliferation in Ca Ski cells. Interestingly, a slight but significant reduction in cellular proliferation (Figure 6C) was observed when Ca Ski cells were knocked down for SNAT1, even in the presence of 2 mM glutamine, suggesting that proliferation induced by E6 and E7 is partially attributable to SNAT1 in Ca Ski cells. Nevertheless, it is possible that other glutamine or glutamate transporters help to compensate for the absence of SNAT1 in the presence of glutamine. As we show in this study, both E6 and E7 oncoproteins upregulate other glutamine transporters such as ASCT2, LAT1, and xCT. Future studies are required to elucidate the joint participation of the different glutamine transporters affected by E6 and E7 in glutamine-dependent proliferation. 

In accordance with these data, ablation of SNAT1 was reported to decrease cell proliferation and migration in melanoma and gastric cancer cell models, evidencing that SNAT1 might play an important role in the maintenance of the malignant phenotype [61,62].

Several studies have reported that SNAT1 is a critical glutamine transporter for cancer cells, the overexpression of which is associated with a poor prognosis [51,62,63]. Our results (Figure 7) demonstrate that SNAT1 is increased in tumors compared with normal tissues, and furthermore, that high SNAT1 expression is associated with poorer overall survival of cervical cancer patients. Similarly, the data from the Human Protein Atlas portal confirm this statement since an increase in the expression of SNAT1 also exhibited a worse prognosis for patients with cervical cancer. Other studies demonstrated that high SNAT1 expression is strongly related to pulmonary metastasis and reduced survival in patients with osteosarcoma [51]. Furthermore, Jing X. et al., demonstrated that high levels of SNAT1 protein are related to invasion, metastasis, and progression of gastric carcinomas and are associated with poor survival of patients with gastric cancer [62].

**Figure 8:**
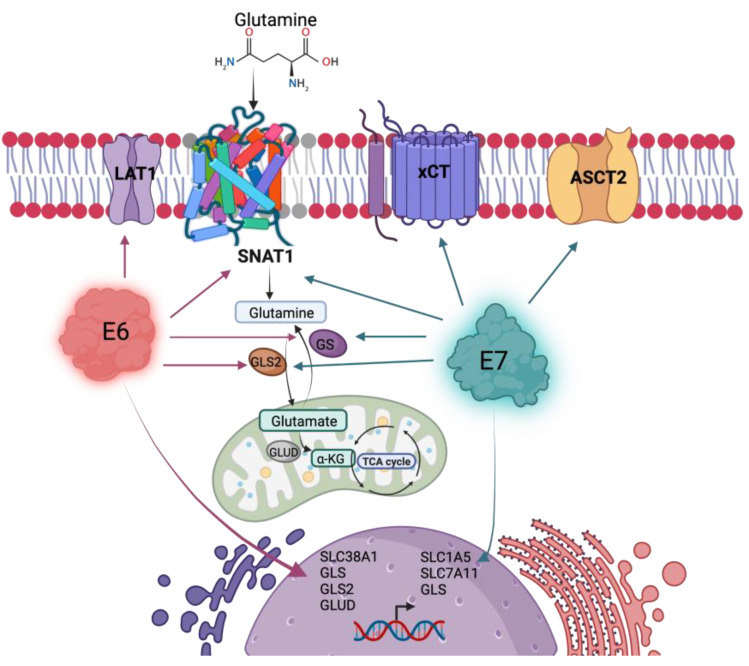
HPV16 E6 and E7 oncoproteins alter the glutamine pathway. Both viral oncoproteins upregulate the protein levels of the SNAT1 transporter, GLS2, and GS. Independently, E6 upregulates LAT1 protein levels and gene expression of SLC38A1, GLS, GLS2, and GLUD; while E7 upregulates ASCT2 and xCT protein levels and gene expression of SLC1A5, SLC7A11, and GLS. Figure created with BioRender.com.

Finally, our results demonstrate that the E6 and E7 oncoproteins promote glutamine uptake and glutamine-dependent cell proliferation, partially attributed to the SNAT1 transporter, although the involvement of other transporters in such events should not be ruled out, as summarized in Figure 8. In addition, we demonstrate that SNAT1 is increased in cervical cancer in relation to the normal cervix and its high expression could be associated with a poor prognosis in patients with cervical cancer. Therefore, understanding the biological mechanisms by which HPV induces an increase in the activity of metabolic pathways, such as glutaminolysis, will eventually allow the development of therapeutic strategies, as well as the identification of biomarkers in HPV-associated cancers.

## Data Availability

Data are contained within the article and Supplementary Materials.

## Supplementary Materials

The following supporting information can be downloaded at: https://www.mdpi.com/article/10.3390/v15020324/s1, Figure S1: The SNAT1 transporter is increased in presence of the HPV16 E6 and E7 oncoproteins; Table S1: Primers used for the amplification of genes analyzed by RT-qPCR.
