# Supplementary material for: HPV16 E6 and E7 Oncoproteins Stimulate the Glutamine Pathway Maintaining Cell Proliferation in a SNAT1-Dependent Fashion
Source: Viruses. 2023 Jan 24;15(2):324. doi: 10.3390/v15020324 (PMC9964736; doi:10.3390/v15020324)

Supplementary Figure S1. The SNAT1 transporter is increased in the presence of HPV16 E6 and E7 oncoproteins. Total protein cell lysates from C-33 A cells harboring EV (black bars), E6 (pink bars) and E7 (green bars) were analyzed by Western blot assay. (A) Representative immunoblots of SNAT1 are shown. As loading control α-tubulin was used. (B) Relative protein levels were obtained from densitometric analysis of immunoblots. (C) Gene expression of SLC38A1 (SNAT1) increase in E7-C-33 A expressing cells. 18S expression gene was used for normalization in qPCR assays. Data from three independent experiments were collected and plotted showing the mean and ±SEM. Student's t-test was performed to analyze statistical differences of E7 and E6 groups compared to the control EV group. ** $p<0.01$, *** $p<0.001$ as indicated.

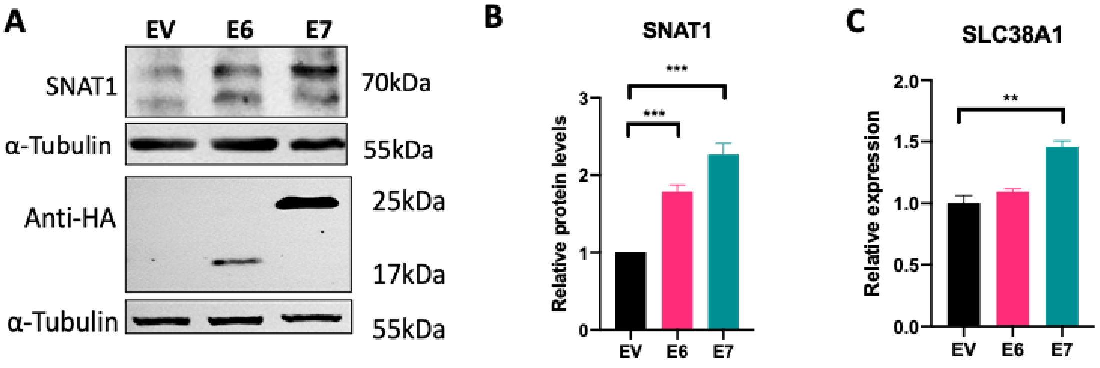

Supplement: Supplementary file 1 [file viruses-15-00324-s001.zip › Supplementary Figure S1.pdf]
