# Supplementary material for: HPV16 E6 and E7 Oncoproteins Stimulate the Glutamine Pathway Maintaining Cell Proliferation in a SNAT1-Dependent Fashion
Source: Viruses. 2023 Jan 24;15(2):324. doi: 10.3390/v15020324 (PMC9964736; doi:10.3390/v15020324)
Supplement: Supplementary file 1 [file viruses-15-00324-s001.zip › Supplementary Table S1.pdf]

Supplementary Table S1. Primers used for the amplification of the genes analyzed by RT-qPCR.

Gene	Primers used for amplification
SNAT1<br>(SLC38A1)	Forward 5’-CACAGACCAGGATGGAGATA-3’<br>Reverse 5’-CTGACCAAGGAGAACAACAC-3’
xCT<br>(SLC7A11)	Forward 5’-CAGTTGCTGGGCTGATTTA-3’<br>Reverse 5’-GAAAGGGCAACCATGAAGA-3’
ASCT2<br>(SLC1A5)	Forward 5`-CTGCCTTTGGGACCTCTTC-3`<br>Reverse 5`-AACGGCTGATGTGCTTGG-3`
LAT1<br>(SLC7A5)	Forward 5`-CCGTGAACTGCTACAGCGT-3`<br>Reverse 5`-CTTCCCGATCTGGACGAAGC-3`
GLS	Forward 5’-ATGATGTGCTGGTCTCCTC-3’<br>Reverse 5’-ATTTATCACTGACTTTACCCTTTG-3’
GLS2	Forward 5’-CCAGTCAGAGAAGGAAACAG-3’<br>Reverse 5’-GAACACAGCTGGAAGTAGAG-3’
GLUD1	Forward 5’-GGATTCTAACTACCACTTGCTC-3’<br>Reverse 5’-GAACGCTCCATTGTGTATGC-3’
18S	Forward 5’-AACCCGTTGAACCCATT-3’<br>Reverse 5’-CCATCCAATCGGTAGTAGCG-3’
